# Effects of Exercise on Cognitive Performance in Older Adults: A Narrative Review of the Evidence, Possible Biological Mechanisms, and Recommendations for Exercise Prescription

**DOI:** 10.1155/2020/1407896

**Published:** 2020-05-14

**Authors:** Adria Quigley, Marilyn MacKay-Lyons, Gail Eskes

**Affiliations:** ^1^Department of Physiotherapy, Dalhousie University, Halifax, NS, Canada; ^2^Department of Medicine, Dalhousie University, Halifax, NS, Canada; ^3^Nova Scotia Health Authority, Halifax, NS, Canada; ^4^Department of Psychiatry, Dalhousie University, Halifax, NS, Canada; ^5^Department of Psychology and Neuroscience, Dalhousie University, Halifax, NS, Canada

## Abstract

Physical activity and exercise have emerged as potential methods to improve brain health among older adults. However, there are currently no physical activity guidelines aimed at improving cognitive function, and the mechanisms underlying these cognitive benefits are poorly understood. The purpose of this narrative review is to present the current evidence regarding the effects of physical activity and exercise on cognition in older adults without cognitive impairment, identify potential mechanisms underlying these effects, and make recommendations for exercise prescription to enhance cognitive performance. The review begins with a summary of evidence of the effect of chronic physical activity and exercise on cognition. Attention then turns to four main biological mechanisms that appear to underlie exercise-induced cognitive improvement, including the upregulation of growth factors and neuroplasticity, inhibition of inflammatory biomarker production, improved vascular function, and hypothalamic-pituitary-adrenal axis regulation. The last section provides an overview of exercise parameters known to optimize cognition in older adults, such as exercise type, frequency, intensity, session duration, and exercise program duration.

## 1. Introduction

In 2017, the number of people aged 60 years or over was 962 million worldwide; this figure is expected to reach nearly 2.1 billion by the year 2050 [1]. This demographic shift toward an older population increases the prevalence and severity of chronic diseases [2]. Dementia is characterized by difficulties with memory, language, thinking, and activities of daily living [3] and can be due to a variety of etiologies, including Alzheimer's pathology and vascular disease [4]. Currently, 35.6 million people worldwide are living with dementia [5]; by the year 2030, that number is expected to double to 75.6 million, making it a major public health priority [6]. Mild cognitive impairment (MCI) is an intermediate stage in the continuum from normal cognition to dementia; it is estimated that 60–65% of people with MCI will develop clinical dementia during their lifetime [7]. Slowing or halting this progression can have implications for quality of life and health care savings. Indeed, if the progression to Alzheimer's disease (the commonest form of dementia) could be delayed by one year, total costs could be reduced by an estimated 113 billion American dollars by the year 2030 [8].

Physical activity and exercise have emerged as potential methods to improve brain health among older adults. However, there are currently no physical activity guidelines aimed at improving cognitive function, and the mechanisms underlying these cognitive benefits are poorly understood. Thus, the main purpose of this narrative review is to present the current evidence regarding the effects of chronic physical activity and exercise on cognition, identify potential mechanisms underlying these effects, and make recommendations for exercise prescription to enhance cognitive performance. Our focus is mainly on older adults over 50 years of age without cognitive impairment. The review begins with a summary of evidence regarding the association between exercise and cognition, adding to older literature reviews such as those by Kirk-Sanchez and McGough and Bherer et al. [9, 10]. Attention then turns to four main biological mechanisms that appear to underlie exercise-induced cognitive improvement among older adults: (i) upregulation of growth factors and neuroplasticity, (ii) inhibition of inflammatory biomarker production, (iii) improved vascular function, and (iv) hypothalamic-pituitary-adrenal (HPA) axis regulation. The last section provides an overview of exercise parameters thought to optimize cognition in older adults.

## 2. Physical Activity and Cognitive Function

Evidence from prospective and cross-sectional studies suggests that physically active persons have a significantly reduced risk of cognitive impairment and dementia. Hamer and Chida [11] conducted a meta-analysis on physical activity and risk of neurodegenerative disease that involved 16 prospective studies of 163,797 participants without dementia. Among participants who were physically active, there was a 28% reduction in risk of dementia and a 45% reduction in Alzheimer's disease [11]. A later meta-analytic review of 21 longitudinal studies of 89,205 adults over the age of 40 also found that higher levels of physical activity was associated with reduced risk of cognitive decline and dementia [12]. Another meta-analysis of 15 prospective studies found that physically active individuals (mostly older adults) reduced their risk of cognitive decline by 38% [13]. Meta-analyses by Daviglus et al. and Beckett et al. each with 9 prospective studies among older adults determined that physically active older adults reduced their risk of developing Alzheimer's disease, relative to their inactive counterparts [14, 15]. The evidence from prospective and cross-sectional research indicates that a physically active lifestyle is associated with a reduction in risk of cognitive impairment later in life.

## 3. Exercise and Cognitive Function among Older Adults: Meta-Analytic Evidence

There is evidence emerging from randomized controlled trials (RCTs) and meta-analyses to suggest that exercise interventions can improve cognition among older adults. The most common experimental intervention in the meta-analyses evaluating the impact of exercise on cognition is aerobic exercise (AE), followed by resistance exercise (RE), a combination of AE and RE, and mind-body exercise (Tai chi, Qigong, dance, or yoga). The meta-analyses are summarized in Table 1 and are discussed below.

Meta-analytic evidence supports the positive effects of AE and RE on cognition among older adults. Colcombe and Kramer determined that there were positive effects of AE and combined (AE and RE) training on cognition in their meta-analysis of 18 RCTs in sedentary older adults [17]. Scherder et al. revealed in their meta-analysis of 8 RCTs that walking improved set-shifting and inhibition in sedentary older adults without cognitive impairment [23]. Northey et al. conducted a meta-analysis of 36 RCTs among older adults and determined that AE, RE, and combined (AE and RE) interventions are all effective in improving cognition in older adults [21]. The most comprehensive meta-analysis to date evaluating cognitive outcomes following exercise interventions revealed a strong effect of AE on executive function among healthy older adults [16]. However, it is important to note that this meta-analysis had a large proportion of female participants and it is thought that exercise may elicit larger improvements in cognition in women than in men [17, 29].

There is also evidence that mind-body exercise can have a positive effect on cognition among older adults. Wu et al. [26] discovered significant improvements in global cognition, cognitive flexibility, working memory, verbal fluency, and learning in their meta-analysis of 32 RCTs among older adults with and without cognitive impairment. Zhang et al. [28] revealed in their meta-analysis of 11 RCTs that mind-body exercise had a significant effect on global cognition, executive function, learning, memory, and language among older adults with and without cognitive impairment. The authors determined that cognitively intact older adults benefitted more from mind-body exercise than those with existing cognitive impairment [28]. Similarly, a 2014 meta-analysis of 11 RCTs by Wayne et al. [25] revealed a robust effect of Tai chi on executive function in older adults without cognitive impairment. Finally, Gothe and Mcauley [19] revealed a moderate effect of yoga compared to controls on measures of attention, processing speed, memory, and executive function in their meta-analysis of 15 RCTs. It is important to note, however, that this meta-analysis was conducted with participants of all ages, and only 6 of the included RCTs were conducted with older adults [19].

In contrast with the those findings, Sanders and colleagues [22] conducted a large meta-analysis of 36 RCTs (23 of which were conducted with healthy older adults) and revealed only a small effect of AE, RE, combined AE and RE, and balance exercise on executive function and memory among healthy older adults. These results may be explained by the exclusion of some studies that did not specify exercise intensity and interventions whose dose parameters were gradually increased (which is recommended by the American College of Sports Medicine guidelines for exercise prescription [30]). Other meta-analyses have also found conflicting evidence on this topic. A Cochrane meta-analytic review by Young et al. [27] of 12 RCTs concluded that there is little evidence that AE of varying doses (for example, three interventions lasted eight weeks and one offered a single exercise class per week) improves cognition in healthy older people. While this meta-analysis was focused on older adults, the most recent included study was from 2012, and more compelling evidence on this topic has been published since then [16, 21]. A 2014 meta-analysis of 25 RCTs on cognition in healthy older adults by Kelly et al. [20] demonstrated beneficial effects of RE versus stretching on reasoning performance and Tai chi on attention and processing speed compared to no exercise, but no effect of AE on cognition in healthy older adults. These findings can be explained by the fact that the authors only included studies conducted between 2002 and 2012, thereby limiting the studies for inclusion [20]. Furthermore, they did not exclude acute or short-term studies, which may have influenced their results [20].

Further conflicting evidence comes from a meta-analysis by Etnier and colleagues [18] who determined that AE and RE have a small overall effect on cognitive performance. However, this study does not adequately report their study selection criteria and exercise dose [18]. Despite this, their analysis of 134 studies revealed that the largest effect sizes were found among adults aged 45–60 years regarding exercise and cognitive performance [18]. Smith et al. published a meta-analysis of the effects of AE on cognitive performance consisting of 29 RCTs that included adults with and without cognitive impairment [24]. Their results revealed modest improvements in attention, processing speed, executive function, and memory in participants randomly assigned to an AE intervention compared to nonaerobic exercising controls [24]. It is worth noting, however, that they also used an inclusion criterion of mean age of 18 years older for their review [24]. Although the majority of the studies they included in the meta-analysis report on older adults, seven of the included studies have substantially younger age ranges, which may not generalize well to older adults [24].

**Figure 1:**
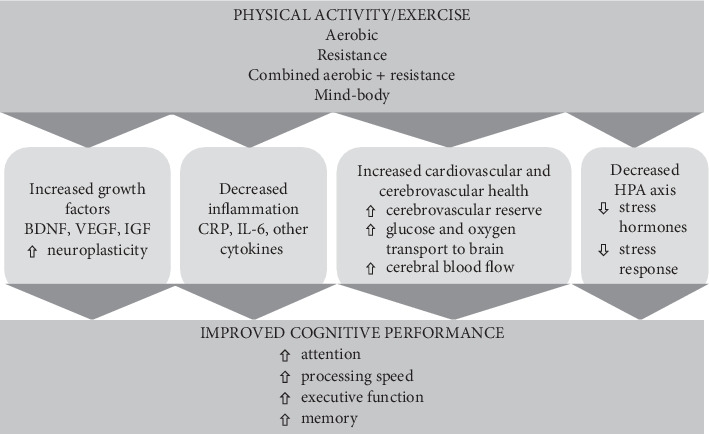
Overview of potential biological mechanisms underlying cognitive gains with physical activity and exercise.

Despite these conflicting reports, 12 of the 13 meta-analyses we reviewed found a beneficial effect of exercise on at least one aspect of cognition albeit some studies have determined only a modest effect. Taken together, this evidence suggests that exercise may have beneficial effects on cognition among older adults. In the RCT exercise literature, there is a lack of standardized reporting of exercise interventions (i.e., frequency, intensity, time, and type), which makes it challenging to determine what constitutes an effective intervention. Future research should address methodological concerns and investigate the optimal exercise dose for improving cognition among older adults. The next section of this review addresses biological mechanisms that may explain exercise-induced improvements in cognition.  Figure 1 provides the overview of potential biological mechanisms underlying cognitive gains with physical activity and exercise.

## 4. Exercise, Neuroplasticity, and Growth Factors

With age, the gray and white matter regions of the human brain begin to atrophy, particularly in the prefrontal cortex and the hippocampus [31]. According to a 2-year longitudinal study using magnetic resonance imaging, older adults without dementia can expect to have 1–2% of hippocampal atrophy per year, while individuals with Alzheimer's disease experience larger volume loss [32]. Other studies have shown that increasing age is associated with lower serum [33, 34] and plasma [35] levels of brain-derived neurotrophic factor (BDNF), a key growth factor in the exercise-brain health interaction, as measured by enzyme-linked immunosorbent assays.

Neuroplasticity, the brain's ability to create and reorganize synaptic connections, appears to be an important mechanism for improved cognition with exercise among adults of all ages. An RCT conducted by Colcombe et al. [36] using functional magnetic resonance imaging revealed aerobically trained older adults demonstrated increased neural activity in the frontal and parietal regions of the brain compared to controls. Similarly, Voss et al.'s [37] RCT revealed improvements in functional connectivity in regions that support the default-mode and frontal executive networks following 12 months of AE training in older adults. A cross-sectional study of 165 healthy older adults found that individuals with higher fitness levels had preserved hippocampal volumes and better performance on a spatial memory task compared to those with low fitness levels [38]. It appears evident that neuroplasticity is underlying some of the improvements in cognition with physical activity. Our focus turns to the role of growth factors such as BDNF, insulin-like growth factor-1 (IGF-1), and vascular endothelial growth factor (VEGF), which play a major role in exercise-induced improvements in cognitive performance among older adults.

High-level evidence indicates that there is a link between exercise interventions and upregulation of growth factors in older adults. A meta-analysis by Dinoff et al. of 29 studies involving AE and RE in 910 healthy adults of all ages revealed an overall increase in BDNF levels following exercise interventions [39]. The authors determined that gender and mean age were not correlated with changes in BDNF levels, indicating that BDNF increases with exercise irrespective of age or gender [39]. It is important to note that the authors identified significant heterogeneity in the included studies regarding study populations, exercise interventions, measurement methods, and study quality [39]. A meta-analysis revealed that AE and combined (AE and RE) training did not significantly increase BDNF levels compared to controls; however, this analysis only included 3 RCTs (all with older adult participants) [16].

There is some evidence that exercise type affects upregulation of specific growth factors. Dinoff et al.'s meta-analysis of AE and RE interventions in healthy adults concluded that AE, but not RE, had a significant effect on peripheral BDNF levels [39]. As well, an RCT of 66 older adults with MCI revealed that an acute bout of AE significantly increased serum BDNF and IGF-1 levels, whereas an acute bout of RE increased only serum IGF levels [40]. Cassilhas et al. conducted an RCT with 62 older adult male participants with two experimental groups (24 weeks of high-intensity RE and moderate-intensity RE), revealing significant improvements in serum IGF-1 levels in both experimental groups compared to controls. From the available literature, it appears that AE interventions tend to increase BDNF levels, while RE interventions increase IGF-1 levels.

While some growth factors appear to increase with exercise, less is known about how this association impacts cognitive performance. A 2014 RCT of 49 older sedentary women revealed that a 16-week program of twice-weekly aerobic, resistance, and motor exercises elevated serum BDNF levels and improved verbal fluency, processing speed, attention, and mental switching performance in exercisers compared to waitlist controls [41]. Importantly, larger effect sizes were found in this study regarding cognitive performance than in similar studies involving single or bimodal forms of exercise [41]. Another RCT involving a 1-year moderate-intensity AE intervention with 90 older adults determined that that a positive relationship existed between age and serum BDNF levels in the AE group, whereby adults over the age of 65 experienced the largest increases in BDNF following the intervention [34]. Importantly, they revealed that increases in serum BDNF and improved executive function in the AE group varied by age, with the oldest individuals reaping the largest benefits to cognitive performance [34]. Despite the findings from Dinoff et al., identifying no association between age and BDNF upregulation, these findings indicate that older adults may experience larger improvements in cognition than younger individuals. Regarding IGF-1 levels, an RCT of 62 older adults revealed significant improvements in both cognitive performance and serum IGF-1 levels following an RE intervention [42]. Stein et al. performed a systematic review of seven randomized studies evaluating the effect of exercise interventions (4 AE, 2 RE, and 1 combined AE and RE) on IGF-1 levels and cognitive performance among older adults, revealing that three of the included studies augmented IGF-1 levels, three remained stable, and one reduced IGF-1 levels with beneficial effects on cognitive performance demonstrated in five of the included studies [43]. These results are explained by the aforementioned hypothesis that IGF-1 levels increase with resistance training; indeed, IGF-1 levels and cognitive performance improved in both RE interventions [42, 44].

Other RCTs have found contrasting results regarding growth factors and exercise. A study with 40 older adults assigned to an AE intervention or control group revealed that serum BDNF, IGF-1, and VEGF levels were not significantly upregulated with exercise and there were no between-group differences in immediate or delayed recall, but IGF-1 levels were related to hippocampal volume changes, and changes in IGF-I were associated with delayed recall performance [45]. An RCT of 33 older adults with MCI revealed that female participants improved on multiple tests of executive function despite a reduction in plasma BDNF levels following a 6-month high-intensity AE intervention while male participants increased plasma IGF-1 levels and improved their performance on an executive function task [29]. A similarly designed RCT by the same authors with glucose-intolerant older adults revealed improvements in executive function, reductions in plasma BDNF levels, and no change in plasma IGF levels [46]. Another RCT comparing 1 year of walking to stretching in healthy elderly participants did not find between-group differences in changes in serum levels of BDNF, IGF-1, or VEGF; however, changes in growth factors of the walking group correlated with improved functional connectivity between the parahippocampal and middle temporal gyrus using functional magnetic resonance imaging [47]. Finally, Erickson and colleagues did not find between-group effects of AE on serum BDNF levels, but they did find that hippocampal volume (which increased in the AE group compared to controls) was associated with higher BDNF levels [48].

Clearly, there are significant discrepancies regarding growth factors as a putative mechanism for improving cognitive performance in older adults. There are known problems with the measurement of growth factors in humans, which could help to explain these contrasting results. For example, Knaepen et al. identified significant blood sampling and biochemical analysis concerns regarding measurement of BDNF levels, including clotting time, temperature storage, and a lack of corrected BDNF levels for the shift in plasma volume with exercise [49]. Similar issues have been identified with the measurement of IGF-1 levels [43]. Furthermore, peripheral BDNF levels are prone to significant diurnal fluctuations due to circadian rhythms [50, 51]. For example, Maass et al. measured serum and plasma BDNF levels eight times during their study and found considerable fluctuations among their participants [45]. Standardized protocols should be implemented and reported by researchers to reduce the measurement error. It is also difficult to determine the extent to which peripheral BDNF levels correlate with central BDNF levels; only one study has found a positive association between peripheral and central BDNF levels, and that study was conducted with people with psychosis [52].

Although there is a considerable discrepancy in the literature, it appears that BDNF levels are increased following AE interventions in people of all ages. Less is known regarding the upregulation of growth factors as a putative mechanism for improved cognitive performance in older adults. High-quality RCTs and meta-analyses that address measurement issues are needed to determine both the effect of exercise on growth factors and the relationship between exercise-induced increases in growth factors and cognitive performance in older adults.

## 5. Inflammation, Exercise, and Cognition

In aging adults, microglia and cytokines stimulate the production of proinflammatory markers, which promote changes in blood vessel permeability, endothelial cell function, and microvascular structure [53], resulting in damage to neurons [54]. Acute and chronic inflammation can facilitate the release of reactive oxygen species and other neurotoxic factors [55]. The aging human hippocampus and basal ganglia have more enzymes involved in inflammatory processes than other brain regions; as such, they tend to be at higher risk of inflammatory damage [56].

As people age, proinflammatory markers such as C-reactive protein (CRP), interleukin (IL)-6 and -1 beta, and tumor necrosis factor alpha (TNF-alpha) increase [53] and are related to cognitive decline [57]. Systemic inflammation is present in chronic conditions such as type 2 diabetes, atherosclerosis [58], multiple sclerosis, and dementia [59]. Studies of older adults and individuals with type 2 diabetes revealed that those with higher levels of inflammation had smaller hippocampi and medial temporal lobes compared to those with low levels of inflammation [60, 61]. A cross-sectional study of 3,298 multiethnic older adults reported that serum levels of IL-6 were negatively associated with the Mini-Mental State Examination scores after adjusting for age, education, and vascular risk factors, leading the authors to conclude that elevated inflammation may have a direct impact on cognition [62]. A longitudinal study of 3,031 healthy older adults found that those with the highest concentrations of IL-6 and CRP had a 24% increased risk of developing cognitive impairment compared to individuals with low inflammation [57].

There is encouraging evidence from a recent systematic review of 13 RCTs that healthy sedentary adults of all ages who participate in AE and RE can reduce inflammatory biomarkers [63]. Stronger effects were found in older adults, with high-intensity AE being the most effective in reducing inflammation [63]. A nonrandomized study involving adults over 60 who participated in 16 weeks of aerobic, resistance, and neuromotor exercise (which includes balance, coordination, agility, gait, and proprioceptive training)demonstrated greater reductions in TNF and IL-6 and increases in peripheral BDNF in the exercise group than in the nonexercising group [64]. Further analyses revealed that exercisers with MCI had significant improvements in executive function and attention [64]. It is thought that AE may release muscle-derived anti-inflammatory substances, thereby reducing the function of proinflammatory cytokines in older human adults [65].

Some promising evidence has emerged indicating that aerobic exercise in particular can reduce inflammatory markers in older adults. While there is evidence that inflammation can have a direct impact on cognition, little is known regarding the mediating role of exercise-induced changes in inflammation on cognitive performance in older adults. Future studies could further investigate the mechanisms underlying the effect of exercise on inflammatory markers and cognition.

## 6. Vascular Health, Exercise, and Cognition

Cardiovascular and cerebrovascular disease risk factors such as hypertension, dyslipidemia, diabetes, and hyperinsulinemia increase the risk of cognitive impairment and dementia [66, 67]. An observational study followed 3,381 adults for 25 years and found that elevated blood pressure and higher fasting blood glucose increased the risk of cognitive impairment and dementia later in life [67]. Consistent with these results, a negative relationship between hypertension and attention, visuospatial, perceptual, memory, learning, psychomotor, and executive performance has been reported in older adults [66]. In the hypertensive brain, decreased vascular blood flow and metabolism in the putamen, globus pallidus, and left hippocampus contribute to higher rates of cognitive impairment in elderly individuals [68, 69]. In older adults, reduced cerebral perfusion related to cardiovascular disease and aging may affect subcortical white matter pathways [70], thereby reducing processing speed and accuracy. Blood vessel stiffening and fibrosis can be contributing factors [71], and free radical production increases with age and cardiovascular disease, all of which can disrupt smooth muscle vasodilatory mechanisms [72]. Age and coronary heart disease also decrease cerebrovascular reactivity to carbon dioxide, thereby increasing the risk of neurological damage [73].

The positive effects of AE on cardiovascular [74–76] and cerebrovascular [77, 78] health among adults of all ages have been well established in the literature. Compared with age-matched sedentary controls, aerobically fit older adults have better cardiac function, including elevated stroke volume, wall thickness, and end diastolic volume [79–81]. Furthermore, chronic AE in older adults results in improved cardiac output, ejection fraction, and left ventricular contractility [80, 82]. A cross-sectional study of 307 participants revealed that healthy men aged 18–79 with better aerobic fitness had 17% higher cerebral blood flow compared to their sedentary counterparts [77].

Some investigators have posited that there may be a link between improved cardiovascular and cerebrovascular function and cognitive performance. Vidoni et al. determined that changes in fitness levels mediated cognitive improvements in older adults who participated in an AE intervention [83]. Brown and colleagues found a significant association between physical fitness, cerebrovascular regulation, and cognitive function in a cross-sectional study of 42 healthy older women [78]. An RCT of older adults revealed that 12 weeks of AE training resulted in higher resting cerebral blood flow in the anterior cingulate region and improved immediate and delayed memory scores compared to controls [84]. Finally, higher fitness levels among female participants of all ages were associated with improved executive function and increased cerebral oxygenation in the frontal areas of the brain compared to women with low fitness levels [85]. Enhanced ability of cerebral blood vessels to respond to chemical, mechanical, or neural demands may be an important mechanism underlying exercise-induced cognitive improvements. Increased blood flow may increase endothelial nitrous oxide synthase expression, which promotes blood vessel vasodilation [86]. Exercise training is also known to enhance oxygen and glucose transport to the brain, thereby increasing cognitive efficiency [87].

Despite the evidence that improved cardiovascular fitness is associated with improved cognition, two meta-analytic reviews have failed to support cardiovascular fitness as a moderator of exercise-induced cognitive gains in older adults [27, 88]. Further evidence to refute the cardiovascular fitness hypothesis is provided by Smiley-Oyen and colleagues, who performed a mediational analysis within their RCT of 57 older adults and revealed improvements in executive function for those who performed AE; however, changes in aerobic fitness were unrelated to changes in executive function [89]. Finally, two other RCTs have found improvements in cognitive performance with RE, AE, and neuromotor exercise, independent of increases in cardiovascular fitness among older adults [90, 91].

There is no question that exercise has beneficial effects on cardiovascular and cerebrovascular function among older adults. Although the cardiovascular hypothesis for exercise-induced changes in cognitive performance in older adults is intuitive, the evidence from meta-analyses and RCTs indicates that this hypothesis alone is unable to explain improvements in cognition with exercise. Is it possible, however, that older adults can experience cognitive benefits with exercise without changes in aerobic fitness. Future research should further elucidate these relationships in order to develop recommendations regarding exercise dose.

## 7. Hypothalamic-Pituitary-Adrenal Axis, Exercise, and Cognition

Another mechanism by which exercise could improve cognitive function is via the HPA axis. As individuals age, their ability to adapt and cope with stress diminishes [92]. Chronic stress can overactivate the HPA axis, which has important implications for glucose tolerance, neuroendocrine, and autonomic functions [93]. Acute stress has known detrimental effects on working memory, interference control, and cognitive flexibility, as evidenced by a recent meta-analysis of 51 experimental studies involving 2,486 adults of all ages [94]. However, the authors found that stress levels did not moderate the effect of cortisol on inhibition and working memory, and studies that utilized cortisol administration did not find an effect on executive functions overall [94]. The authors found that participant age did not moderate stress effects on executive functions; however, only three of their included studies were conducted with older adults, therefore it is unlikely that these results can be applied to older adults. Indeed, there is longitudinal and cross-sectional evidence indicating that older adults with higher rates of chronic stress are 2.7 times more likely to develop Alzheimer's disease [95], and higher cortisol levels are associated with worsening cognition among older adults [96–98]. In contrast, a longitudinal study of 52 older adults found that higher cortisol levels were associated with slower cognitive decline in those with MCI but not in those with normal cognition [99]. Cortisol is known to be susceptible to considerable fluctuations throughout the day [100], whereby cortisol levels increase upon waking, then steadily decline throughout the day [101]. This difficulty in measuring cortisol levels may explain some of the discrepancies in the aforementioned studies.

Exercise can downgrade the stress response in individuals of all ages by regulating the release of catecholamines and cortisol [102]. During exercise, the HPA axis is activated, and there is a subsequent increase in tissue sensitivity to circulating glucocorticoids [103]. This action buffers the inflammatory processes in the muscle and cytokine production, thereby reducing exercise-induced inflammation [103]. A review of 8 interventional studies evaluating chronic RE and combined interventions in older adults revealed a reduction in serum cortisol over time in four studies; however, many of the included studies lacked a control group, were not randomized, and measured only morning cortisol levels [92].

There is evidence that mind-body exercise has a downregulating effect on the sympathetic nervous system and the HPA axis [104]. A systematic review of 25 RCTs in individuals with and without chronic diseases revealed that yoga interventions improved the sympathetic nervous system and HPA regulation (using measures of cortisol, heart rate, and blood pressure) compared to controls; however, this review was not conducted on older adults [102]. A meta-analytic review of 40 interventional studies (the majority of participants were older adults) found that Tai chi has positive effects on both anxiety and depression [105]. A cross-sectional study of 42 middle-aged and older adults revealed that people who practiced Tai chi and yoga demonstrated significant improvements in mental health outcomes compared to those who performed AE [106].

Four meta-analyses have found beneficial effects of mind-body exercise on cognition among older adults [21, 25, 26, 28]. Although the mechanism is currently unknown, it is possible that yoga and Tai chi can contribute to dominance of the parasympathetic nervous system [104, 107], which may improve cognitive performance [108]. An RCT of 118 older adults investigated this theory with an 8-week yoga intervention, after which yoga participants demonstrated improved executive function and an attenuated cortisol response compared to those in the control group [108]. Importantly, the change in self-reported anxiety and cortisol levels predicted performance on the executive function tasks, indicating that downgrading the HPA response is a potential mechanism by which mind-body exercise improves cognition among older adults [108]. This is the only known study to evaluate the potential of the HPA axis as a mediator in cognitive performance improvements among older adults to date.

Mind-body exercise is an effective intervention for improving cognition among older adults, possibly by reducing stress levels and restoring parasympathetic-sympathetic nervous system balance. Though this evidence is encouraging, this topic has only begun to be studied. Mediational RCTs and meta-analyses examining these outcomes are needed to examine the relationship between mind-body exercise, the HPA axis, and cognitive function among older adults.

## 8. Exercise Prescriptions to Improve Cognitive Health in Older Adults

Currently, there are no exercise guidelines that specifically target cognitive gains in older adults, but clues can be gleaned from the literature on exercise-induced cognitive change. Factors related to an exercise program—exercise type, intensity, session duration and frequency, and program duration—may determine the extent to which exercise impacts cognition. In terms of *exercise type*, most investigations have focused on the effectiveness of AE [16, 17, 21, 23, 24] although RE [16, 20, 21] and mind-body exercise [25, 26, 28] such as Tai chi and yoga have shown to be effective in improving cognition among older adults.

Some trials have reported beneficial effects of combined AE and RE exercise [41, 64, 109]. Colcombe and Kramer concluded in their meta-analysis of 18 RCTs in sedentary older adults that combined RE and AE yielded larger effects on cognition compared to AE alone or no exercise [17]. Similarly, Barha and colleagues discovered in their meta-analysis of healthy older adults that combined training benefitted global cognitive function and episodic memory more than AE and RE alone [16]. We conclude that single exercise modes are effective in inducing improvements in cognition among older adults, but combined modes may offer additional cognitive benefits. Future RCTs could evaluate the effect of various subtypes of exercise on cognitive performance among older adults, including AE (i.e., upper limb versus lower limbs, concentric versus eccentric, and aquatic versus land-based), RE (i.e., progressive resistance exercise and plyometric training), and neuromotor exercise (i.e., agility, balance, and proprioceptive training).

Exercise *frequency* also appears to be an important predictor of cognition. Northey et al. performed a moderator analysis within their meta-analysis and determined that more frequent (5–7 sessions per week) exercise of all types was more beneficial for cognition than less frequent (≤2) or moderately-frequent (3–4) exercise in older adults [21]. These findings are disputed by those of Sanders and colleagues, who revealed that exercise frequency (and in fact, all dose parameters) did not predict changes in cognition among older adults [22]. However, as previously mentioned, Sanders and colleagues excluded several studies where the exercise dose was increased over time [22]. As such, their results may not be applicable to the majority of exercise programs where it is recommended to progressively increase the exercise dose over time. We therefore conclude that older adults may experience cognitive benefits from exercise 5–7 times per week.

Exercise *intensity* may be a significant predictor of cognitive benefit [38, 110]. In their meta-analysis of 36 RCTs, Northey et al. performed a moderator analysis of exercise intensity and determined that high- and moderate-intensity exercise of all types were superior to low-intensity exercise in terms of cognition in older adults; however, the effect sizes were small [21]. In contrast, other evidence indicates that improvements in fitness levels are not required to improve cognition among older adults [17, 27, 89]. Moreover, improvements in cognition have been obtained using low-intensity exercise, such as Tai chi [25, 26, 28] and yoga [19]. As such, we conclude that exercise at any intensity may benefit cognition in older adults.

Longitudinal studies have reported that *exercise session duration* may be an important predictor of cognition among older adults [14, 15]. This is supported by evidence from the meta-analysis by Northey et al., who determined that sessions lasting 45 minutes or longer were more beneficial for cognitive performance than shorter sessions [21]. An RCT conducted with four groups of older adults (controls, 50%, 100%, or 150% of the recommended dose of 150 minutes per week) who performed AE of progressive intensities for 26 weeks revealed that a change in fitness mediated the effect of session duration on visuospatial performance [83]. A dose response was apparent, with longer session durations enhancing improvements in visuospatial function [83]. We conclude that exercise sessions for older adults should ideally last 45 minutes or longer.

In relation to *exercise program duration*, some investigators argue that cognitive improvements may not be apparent until 6 months [29] or a year of exercise training [111], while other studies have reported positive results in as little as 8 [112], 12 [84, 113], or 16 weeks [41]. A 2018 systematic review of 98 RCTs of older adults with and without cognitive impairment determined in their analysis of optimal exercise dose that exercising for at least 52 total hours was associated with improved cognition [114]. Furthermore, their bivariate correlation analysis revealed that the most important predictor of improved cognition was total intervention duration [114]. In contrast, the meta-analysis by Northey and colleagues revealed similar effect sizes for programs of short (4–12 weeks), medium (13–26 weeks), and long (>26 weeks) durations [21], while Asteasu and colleagues made a similar observation in their systematic review [115]. It is important to note that the effect of exercise program duration on cognition could be influenced by the frequency of exercise sessions and exercise session duration, as discussed by Sanders and colleagues; therefore, it may be more valuable to look at the total exercise dose. As such, we conclude that exercise interventions for older adults have a total duration of at least 52 hours. Future studies should ensure that the total exercise dose is explicitly stated and is equal between comparison groups.

## 9. Conclusion and Areas for Future Inquiry

The present paper has provided a review of the literature regarding the effect of exercise on cognition and an overview of the likely biological mechanisms underlying this interaction. The evidence indicates that physical activity, notably multimodal and mind-body exercise, offers benefits to cognition in older individuals. The mechanisms underlying these benefits are numerous and overlapping. However, there are many gaps in the literature, such as a lack of high-quality studies evaluating moderators of cognitive function among older adults. Future research using is needed to determine optimal exercise parameters and exercise subtypes aimed at improving cognition in this population.

## Figures and Tables

**Table 1:** Meta-analyses of RCTs evaluating the impact of exercise interventions on cognition.

First author, year		# of participants	Population (*s*)	Other selection criteria	Experimental exercise (# of studies)	Exercise dose	Control treatment	Meta-analysis results
Barha et al. (2017) [16]		39 RCTs (5,256 participants)	Healthy older adults 45+ years	(i) Mind-body exercise trials excluded (ii) Studies evaluating global cognition only excluded	AE (*n* = 19) RE (*n* = 9) Combined AE + RE (*n* = 13)	Frequency: 1–5 sessions/week Intensity: variable Duration:8–52 weeks	Active and inactive controls	(i) AE improved executive functioning relative to controls (*g*=2.064) (ii) AE interventions benefitted global cognitive function and executive functions more than RE (iii) Combined training benefitted global cognitive function and episodic memory more than AE and RE

Colcombe and Kramer (2003) [17]		18 RCTs 197 participants	Older adults, 55–80 years	(i) Date range 1966–2001 (ii) Measure of aerobic fitness	AE (49% of participants) or combined AE + RE (51% of participants)	Frequency: NR Intensity: NR Duration: 1–3 months (38%), 4–6 months (36%) 6+ months (26.7%)	NR	(i) Exercise had the greatest effect on executive function (*g*=0.68) (ii) Combined training produced larger improvements in cognition than AE alone (*g*=0.59 vs. 0.41) (iii) Long-term training associated with largest improvements in cognition (*g*=0.674)

Etnier et al. (1997) [18]		134 studies (17 clinical trials) 420 participants	All ages (exact ages NR)	(i) NR	AE and RE (numbers NR)	NR	NR	(i) Moderate effect of chronic exercise on cognition (*g*=0.33) (ii) Effect of clinical trials on cognition (*g*=0.18) (iii) Exercise session duration, frequency, and program duration were not significant moderators of cognition

Gothe and Mcauley (2015) [19]		22 (15 RCTs) 2,012 participants	All ages (mean age 62 years)	(i) Objective measure of cognitive function	Hatha yoga (*n* = 8) Iyengar yoga (*n* = 3) Integrated yoga therapy (*n* = 1), Sahaj yoga (*n* = 1)	Frequency: 1–5 sessions/wk Session duration: 45–120 mins Duration:1–6 months	Active and inactive	(i) Yoga had a moderate effect (*g*=0.33) on cognition (ii) Largest effect on attention and processing speed (*g*=0.29) followed by executive function (*g*=0.27) and memory (*g*=0.18)

Kelly et al. (2014) [20]		25 RCTs 2,217 participants	Healthy older adults with no cognitive impairment 50+ years	(i) Cardiovascular disease, other medical, psychiatric, or neurological conditions were excluded (ii) Date range 2002–2012	AE (*n* = 19), RE (*n* = 7), Tai chi (*n* = 3)	Frequency: 1–3 sessions/wk Intensity: variable Total duration: single bout to 1 year	No exercise, nonaerobic exercise, education, social or mental activities	(i) No improvements in AE vs stretching/toning on cognition (ii) Significant improvements for RE vs. stretching/toning in reasoning (*p* < 0.005) and Tai chi vs no exercise in attention (*p* < 0.001) and processing speed (*p* < 0.00001)

Northey et al. (2018) [21]		36 RCTs 2,748 participants	Older adults with and without cognitive impairment 50+ years	(i) Neurological and mental health populations excluded (ii) Unsupervised exercise interventions excluded (iii) Interventions <4 weeks excluded	AE (*n* = 18), RE (*n* = 13, multicomponent (*n* = 10), Tai chi (*n* = 4), yoga (*n* = 2)	Frequency: 1–5 sessions/wk Session duration: 20–90 min Intensity: variable, many NR Total duration: 6–52 weeks	Active (stretching, balance and tone, sham cognitive training, health education) and inactive (social interaction), no contact	(i) Improved cognitive function (SMD = 0.29; *p* < 0.01) with exercise of all types (ii) AE, RE, combined, and Tai chi interventions had significant effect estimates (*p* < 0.01) (iii) Session duration >45 min ≤ 60 min associated with improved cognition (*p* < 0.01) (iv) Moderate (*p* < 0.02) and vigorous (*p* < 0.01) intensity exercise (not low-intensity) associated with improved cognition

Sanders et al. (2019) [22]		36 RCTs 2,007 participants	Older adults with and without cognitive impairment 50+ years	(i) Treatment duration >4 weeks (ii) Excluded if did not specify exercise intensity (iii) Excluded studies if dose parameters were gradually increased	AE (*n* = 21) RE (*n* = 18) Multicomponent (*n* = 10) Balance (*n* = 2)	Frequency: 1–5 sessions/wk (mean = 2.62) Program duration: 4–52 weeks (mean = 22.3 weeks) Mean total dose: 2720 mins	Active and passive controls	(i) Small positive effect of exercise on executive function (*d* = 0.27) and memory (*d* = 0.24) in healthy older adults (ii) Exercise dose (type, session, duration, program duration, frequency, intensity) did not predict changes in cognition (iii) Shorter exercise sessions and higher frequency sessions predicted larger effects in those with cognitive impairment

Scherder et al. (2014) [23]		8 RCTs 642 participants	Sedentary healthy older adults 55+ years and older adults with cognitive impairment	(i) Published in English	AE (*n* = 8)	Frequency:1–7 sessions/wk Session duration: 30–60 mins Program duration: 4 weeks–1 year	Active and passive controls	(i) Walking improved set-shifting and inhibition in sedentary older adults without cognitive impairment (*d* = 0.36) (ii) No effect of walking on executive function among older adults with cognitive impairment (*d* = 0.14)

Smith et al. (2010) [24]		29 RCTs 2,049 participants	Healthy adults and MCI Mean age 18+ years 23 RCTs with older adults	(i) Treatment duration: >1 month	Supervised AE (*n* = 29)	Frequency: 1–5 sessions/wk Intensity: variable, many NR Duration: 8–72 weeks	Nonaerobic exercise, waitlist, education, stretching, social activities	(i) Modest improvements in attention and processing speed (*g*=0.158), executive function (*g*=0.123) (ii) Exercise intensity and duration did not moderate effects on memory (iii) Older adults demonstrated larger improvements in working memory compared to younger participants

Wayne et al. (2014) [25]		11 RCTs 2,553 participants	Older adults with and without cognitive impairment Mean age 60 years, except 1 study with adults	(i) Measure of cognitive function	Tai chi (*n* = 11)	Frequency: 1–4 sessions/week Intensity: variable, many NR Duration: 10 weeks–1 year	Active and nonactive controls	(i) Large effect of Tai chi vs no exercise on executive function (*g*=0.90) and moderate effect vs exercise controls (*g*=0.51) in healthy older adults

Wu et al. (2018) [26]		32 RCTs 3,624 participants	Older adults with and without cognitive impairment aged 55–80 years	(i) Measure of cognitive function	Tai chi (*n* = 18) Yoga (*n* = 8) Dance (*n* = 6)	Frequency: 1–4 sessions/week Intensity: NR Session duration: 20–60 mins/session, Duration: 2–48 wks	Active and nonactive controls	(i) Improvements in global cognition compared with controls (mean difference = 0.92), particularly cognitive flexibility, working memory, verbal fluency, and learning (ii) Moderate dose (60–120 mins per week) significantly improved global cognition compared to controls

Young et al. (2015) [27]		12 RCTs 754 participants	Healthy older adults >55 years	(i) Measure of CV fitness	AE (*n* = 12)	Frequency: 1–5 sessions per week Intensity: variable Duration: 8–26 weeks	No treatment, nonaerobic exercise, social or mental activities	(i) No benefit of AE vs active or inactive controls on any of the 11 cognitive domains (ii) Incresed fitness did not coincide with improvements in cognition.

Zhang et al. (2018) [28]		19 RCTs 2,539 participants	60+ years with and without cognitive impairment	(i) Measure of cognitive function (ii) Articles in English or Chinese	Tai chi (*n* = 12) Yoga (*n* = 4) Qigong (*n* = 2) Pilates (*n* = 1)	Frequency: 1–7 sessions/wk Session duration: 20–120 min Intensity: NR, Duration: 8–52 weeks.	Active or passive controls	(i) Benefits global cognition (*g*=0.23), executive functions (*g*=0.25–0.65), learning and memory (*g*=0.37–0.49), and language (*g*=0.35) (ii) Mind-body exercise more effective for older adults without cognitive impairment (iii) Total training time predictor of global cognition, executive function, and language

RCT, randomized controlled trial; MCI, mild cognitive impairment; NR, not reported; min, minutes; AE, aerobic exercise; RE, resistance exercise.
